# Unanticipated prognosis of differential thyroid cancer patients with T0 stage: analysis of the SEER database 2004-2013

**DOI:** 10.18632/oncotarget.19988

**Published:** 2017-08-07

**Authors:** Chunping Liu, Jie Ming, Wen Zeng, Shuntao Wang, Yiquan Xiong, Qiuyang Zhao, Xingjie Yin, Zeming Liu, Tao Huang

**Affiliations:** ^1^ Department of Breast and Thyroid Surgery, Union Hospital, Tongji Medical College, Huazhong University of Science and Technology, Wuhan, China; ^2^ Department of Ophthalmology, Zhongnan Hospital, Wuhan University, Wuhan, China

**Keywords:** differential thyroid cancer, T0 stage, prognosis, SEER

## Abstract

The prognosis of T0 stage differentiated thyroid cancer (DTC) remains unclear. This study aimed to investigate the prognosis of T0 stage DTC patients to provide a new perspective on treatment guidelines for these patients. We investigated a large cohort of DTC patients from the Surveillance, Epidemiology, and End Results (SEER) database between 2004 and 2013. Patient survival curves were examined by Kaplan-Meier analyses with log-rank tests and Cox proportional hazards regression analyses. In the study cohort, the rate of cancer-specific mortality per 1000 person-years for T0 was higher than T1–T3, but lower than T4. The all-cause mortality for T0 patients was higher than all other stages (T1–T4). Multivariate Cox regression modeling showed that T0 had a significant risk for cancer-specific mortality when compared to T1 and T4, but not T2 or T3, after adjustment for other risk factors. For all-cause mortality, T0 showed a significant risk for all-cause mortality when compared to T4, but not T1–T3 stage patients. Similar results were obtained after matching for influential factors using propensity scored matching analysis. The unanticipated prognosis of T0 stage DTC patients was found to be not better than of other stage DTC patients, providing new implications for the treatment of T0 stage DTC patients.

## INTRODUCTION

The incidence of thyroid cancer has risen rapidly in recent decades [1-4]. The follicular cells give rise to two main forms of differentiated thyroid cancer (DTC): papillary thyroid carcinoma and follicular thyroid carcinoma. DTC accounts for approximately 86% of all thyroid carcinomas [1, 5].

The current American Joint Committee on Cancer (AJCC) staging systems: TNM system (versions 6.0 and 7.0) are prognostic systems that predict DTC-specific mortality [6]. These systems are considered gold standards to stratify cancer patients into different risk groups. T0 patients are defined as not having evidence of a primary tumor, according to the TNM system, in both versions 6 and 7. A diagnosis of T0 stage DTC would be confirmed by Fine Needle Aspiration (FNA) biopsy (histological and cytological confirmation) or radioactive iodine uptake test in metastasis sites such as the brain, bone, and lung [7].

The Surveillance, Epidemiology, and End Results (SEER) program of the National Cancer Institute (NCI) is the largest publicly available source of data on cancer incidence and survival [8, 9]. There are few investigations focusing on the prognosis and treatment of T0 stage DTC patients. In a previous study, T0 patients were considered to have a low mortality and had less aggressive treatment as compared to T1–T3 patients [10]. In the present study, we evaluated the prognosis of T0 stage DTC patients as compared to T1 to T4 stage patients, based on SEER data from patients diagnosed between 2004–2013.

## RESULTS

### Demographic and clinical features

A total of 94092 patients who had definite T stage DTC, according to AJCC versions 6 and 7, were included in this study. The distribution of patients by stage were the following: 180 patients were T0, 55615 were T1, 15613 were T2 stage, 17529 were T3, and 3669 were T4 stage. The study patients’ mean age and survival in months for the different T stages are shown in Table 1. T0 patients had significantly shorter survival months than patients with other stages.

**Table 1:** Characteristics for patients with T0-T4

Covariate	level	T stages					*P* value
		T0 (n=180,%)	T1 (n=55615,%)	T2 (n=15613,%)	T3 (n=17529,%)	T4 (n=3669,%)	
Age		54.87±18.73	49.64±14.37	46.22±15.94	49.03±16.41	57.11±17.65	<0.001
Sex	Female	104 (57.8)	45049 (81.0)	11721 (75.1)	12129 (69.2)	2406 (65.6)	<0.001
	Male	76 (42.2)	10566 (19.0)	3892 (24.9)	5400 (30.8)	1263 (34.4)	
Race	White	146 (81.1)	46207 (40.0)	12620 (80.8)	13888 (79.2)	2891 (78.8)	<0.001
	Black	20 (11.1)	3442 (6.2)	1152 (73.8)	1158 (6.6)	192 (5.2)	
	Other	14 (7.8)	5295 (9.5)	1639 (10.5)	2282 (13.0)	563 (15.3)	
Tumor size			9.15±5.84	29.06±5.76	35.95±34.53	36.14±27.94	<0.001
N-stage	N0	30 (16.7)	48296 (86.8)	12370 (79.2)	10479 (59.8)	1353 (36.9)	<0.001
	N1	132 (73.3)	6618 (11.9)	2865 (18.4)	6529 (37.2)	1869 (50.9)	
M-stage	M0	135 (75.0)	55445 (99.7)	15463 (00.0)	17124 (97.7)	3187 (86.9)	<0.001
	M1	45 (25.0)	170 (0.3)	150 (1.0)	405 (2.3)	482 (13.1)	
Histologic subtype	Papillary	159 (88.3)	54362 (97.7)	13480 (86.3)	15825 (90.3)	3476 (84.7)	<0.001
	Follicular	21 (11.7)	1250 (0.3)	2133 (13.7)	1703 (9.7)	193 (5.3)	
Multifocality	No		34596 (62.2)	9413 (60.3)	8776 (50.1)	1737 (47.3)	<0.001
	Yes		20463 (36.3)	5972 (38.2)	8360 (47.7)	1699 (46.3)	
Radiation	None or refused	89 (49.4)	33904 (61.0)	5484 (35.1)	5017 (28.6)	1017 (27.7)	<0.001
	External beam radiation therapy	11 (6.1)	583 (1.0)	303 (1.9)	433 (2.5)	393 (10.7)	
	Radioactive I-131 ablation	74 (41.1)	20038 (36.0)	9451 (60.5)	11587 (66.1)	2170 (59.1)	
Surgery	Biopsy	56 (31.1)	565 (1.0)	270 (17.3)	136 (0.8)	232 (6.3)	<0.001
	Lobectomy	11 (6.1)	9994 (18.0)	1758 (11.2)	1267 (7.2)	220 (6.0)	
	Subtotal or near-total thyroidectomy	4 (2.2)	2192 (39.4)	568 (3.6)	503 (2.9)	145 (4.0)	
	Total thyroidectomy	105 (58.3)	42417 (76.3)	12869 (82.4)	15488 (88.4)	3024 (82.4)	
Survival months		40.47±30.71	49.00±33.48	51.05±34.20	47.77±33.18	47.63±34.89	<0.001

### Cancer specific mortality and all-cause mortality for different stages of DTC

In the study cohort, the rate of cancer-specific mortality, per 1000 person-years, for T0, T1, T2, T3, and T4 stage were 14.83 (95% CI, 7.71–28.50), 0.38 (95% CI, 0.31–0.47), 1.11 (95% CI, 0.89–1.40), 3.40 (95% CI, 2.99–3.86) and 35.70 (95% CI, 32.76–38.91), respectively (Table 2). The all-cause mortality, per 1000 person-years, in patients with T0, T1, T2, T3 and T4 stage were 59.31 (95% CI, 42.78–82.22), 7.68 (95% CI, 7.32–8.04), 8.52 (95% CI, 7.85–9.25), 12.58 (95% CI, 11.78–13.44) and 58.43 (95% CI, 54.63–62.49).

**Table 2:** Hazard ratios of stages for the cancer specific mortality and all cause mortality of DTC

T stage	DTC mortality, no.	%	DTC mortality per 1,000 person-years	95% CI	All cause mortality, no.	%	All cause mortality per 1,000 person-years	95% CI
T0	10	5.56	14.83	7.71-28.50	39	21.7	59.31	42.78-82.22
T1	91	0.16	0.38	0.31-0.47	1811	3.26	7.68	7.32-8.04
T2	77	0.49	1.11	0.89-1.40	576	3.69	8.52	7.85-9.25
T3	243	1.38	3.40	2.99-3.86	891	5.08	12.58	11.78-13.44
T4	556	15.2	35.70	32.76-38.91	905	24.67	58.43	54.63-62.49

DTC: differential thyroid cancer

### Risk factors for thyroid cancer-specific mortality and all-cause mortality

Univariate Cox regression analyses showed that age, male sex, race, TNM stage, follicular subtype, and radiation and surgery approach were significant risk factors of cancer-specific mortality. In the multivariate Cox regression model, T0 stage showed significant higher risk for cancer-specific mortality as compared to T1 and lower risk for T4, but not to T2 and T3 after adjustment of influential risk factors (Table 3). For the all-cause mortality, univariate Cox regression analyses showed that age, male sex, race, TNM stage, follicular subtype, and radiation and surgery approach were significant risk factors. Multivariate Cox regression analysis determined that T0 stage showed significant lower risk for all-cause mortality compared to T4 stage patients, but not T1–T3 stage patients (Table 3).

**Table 3:** Risk factors for survival: outcome of differentia thyroid cancer specific mortality and all-cause mortality

Covariate	Level	Thyroid cancer specific mortality				All cause mortality			
		Univariate Cox regression		Multivariate Cox regression		Univariate Cox regression		Multivariate Cox regression	
		**Hazard Ratio (95% CI)**	**p-value**	**Hazard Ratio (95% CI)**	**p-value**	**Hazard Ratio (95% CI)**	**p-value**	**Hazard Ratio (95% CI)**	**p-value**
Age		1.096 (1.091-1.101)	<0.001	1.063 (1.057-1.068)	<0.001	1.086 (1.084-1.089)	<0.001	1.073 (1.070-1.075)	<0.001
Sex	Female	ref		ref		ref		ref	
	Male	2.765 (2.437-3.137)	0.005	1.223 (1.061-1.409)	0.006	2.419 (2.275-2.572)	<0.001	1.598 (1.496-1.707)	<0.001
Race	White	ref		ref		ref		ref	
	Black	0.993 (0.764-1.291)	0.958	1.144 (0.868-1.508)	0.340	1.267 (1.134-1.416)	<0.001	1.358 (1.208-1.526)	<0.001
	Other	1.372 (1.114-1.648)	0.001	0.978 (0.798-1.198)	0.827	0.865 (0.778-0.962)	0.008	0.797 (0.711-0.893)	<0.001
T-stage	T0	ref		ref		ref		ref	
	T1	0.026 (0.013-0.050)	<0.001	0.193 (0.092-0.404)	<0.001	0.125 (0.091-0.171)	<0.001	0.748 (0.521-1.073)	0.115
	T2	0.076 (0.039-0.146)	<0.001	0.522 (0.248-1.097)	0.086	0.136 (0.098-0.188)	<0.001	0.873 (0.6061.259)	0.468
	T3	0.118 (0.118-0.418)	<0.001	1.172 (0.572-2.401)	0.664	0.200 (0.145-0.275)	<0.001	1.001 (0.697-1.437)	0.997
	T4	2.456 (1.314-4.590)	0.005	4.628 (2.282-9.389)	<0.001	0.970 (0.704-1.336)	0.850	2.223 (1.552-3.185)	<0.001
N stage	N0	ref		ref		ref		ref	
	N1	4.852 (4.245-5.545)	<0.001	1.896 (1.623-2.216)	<0.001	1.626 (1.516-1.744)	<0.001	1.452 (1.336-1.579)	<0.001
M-stage	M0	ref		ref		ref		ref	
	M1	48.240 (42.273-55.049)	<0.001	5.865 (4.958-6.939)	<0.001	12.981 (11.826-14.248)	<0.001	3.453 (3.070-3.885)	<0.001
Histologic subtype	Papillary	ref	ref			ref		ref	
	Follicular	2.804 (2.355-3.339)	<0.001	1.494 (1.217-1.833)	<0.001	1.730 (1.565-1.911)	<0.001	1.157 (1.034-1.294)	0.011
Radiation	None or refused	ref		ref		ref		ref	
	Radiation Beam or Rdioactive implants	16.802 (14.194-19.889)	<0.001	2.326 (1.904-2.841)	<0.001	3.773 (3.362-4.234)	<0.001	1.282 (1.119-1.467)	<0.001
	Radioisotopes or Radiation beam+ isotopes/implants	1.100 (0.952-1.271)	0.197	0.777 (0.656-0.919)	0.003	0.650 (0.610-0.694)	<0.001	0.0683 (0.634-0.736)	<0.001
Surgery	Biopsy	ref		ref		ref		ref	
	Lobectomy	0.031 (0.024-0.041)	<0.001	0.480 (0.352-0.653)	<0.001	0.081 (0.072-0.092)	<0.001	0.317 (0.275-0.365)	<0.001
	Subtotal or near-total thyroidectomy	0.067(0.049-0.091)	<0.001	0.553 (0.382-0.802)	0.002	0.088 (0.074-0.103)	<0.001	0.323 (0.269-0.388)	<0.001
	Total thyroidectomy	0.044 (0.038-0.053)	<0.001	0.444 (0.352-0.559)	<0.001	0.067 (0.061-0.074)	<0.001	0.297(0.262-0.336)	<0.001

### Adjusting for patient characteristics using propensity score matching

**Figure 1:**
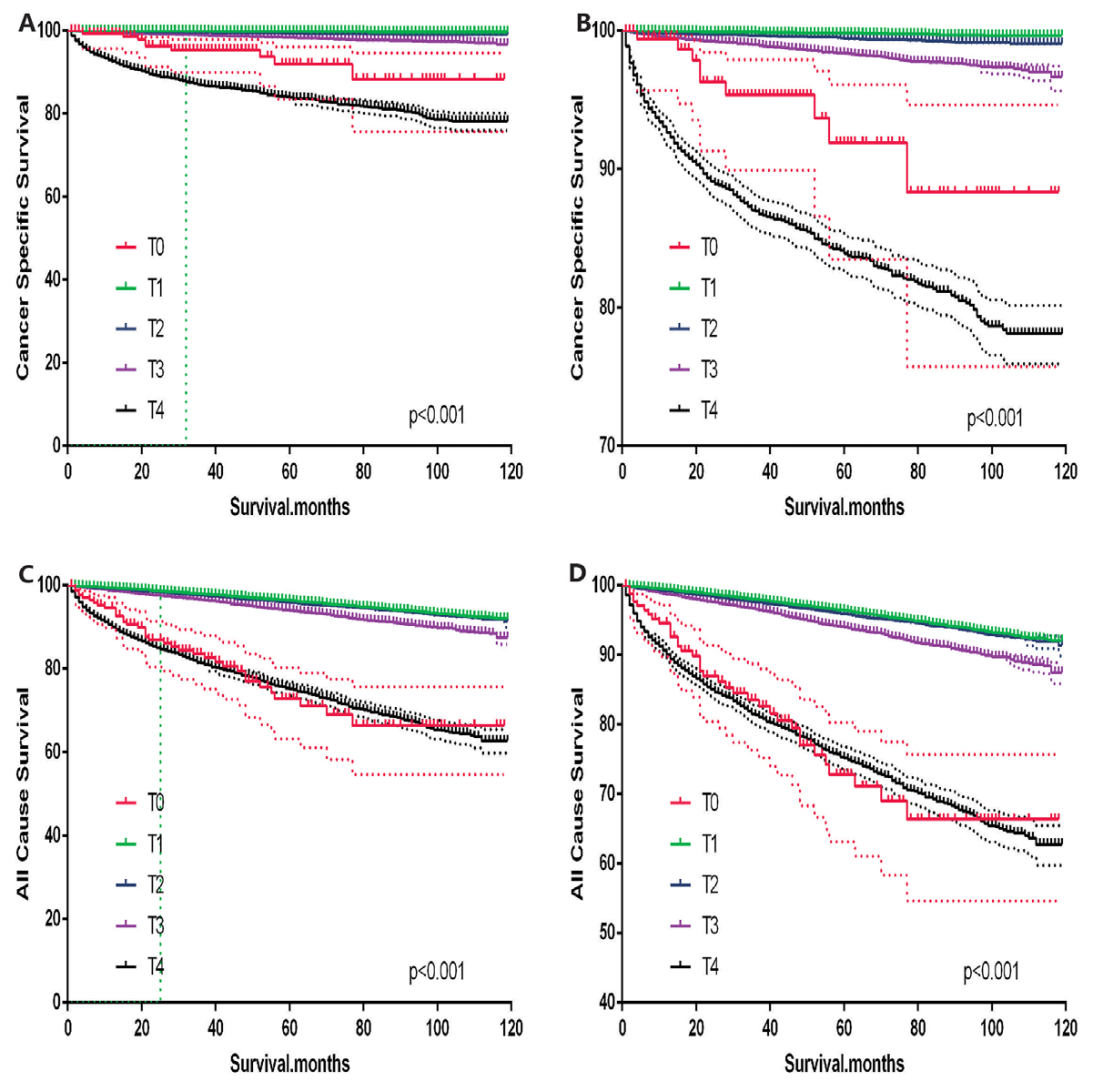
Kaplan Meier curves among patients stratified by T-stage for cancer-specific mortality **(A, B)** Log rank test p < 0.0001) and all cause mortality **(C, D)** Log rank test p < 0.0001).

**Figure 2:**
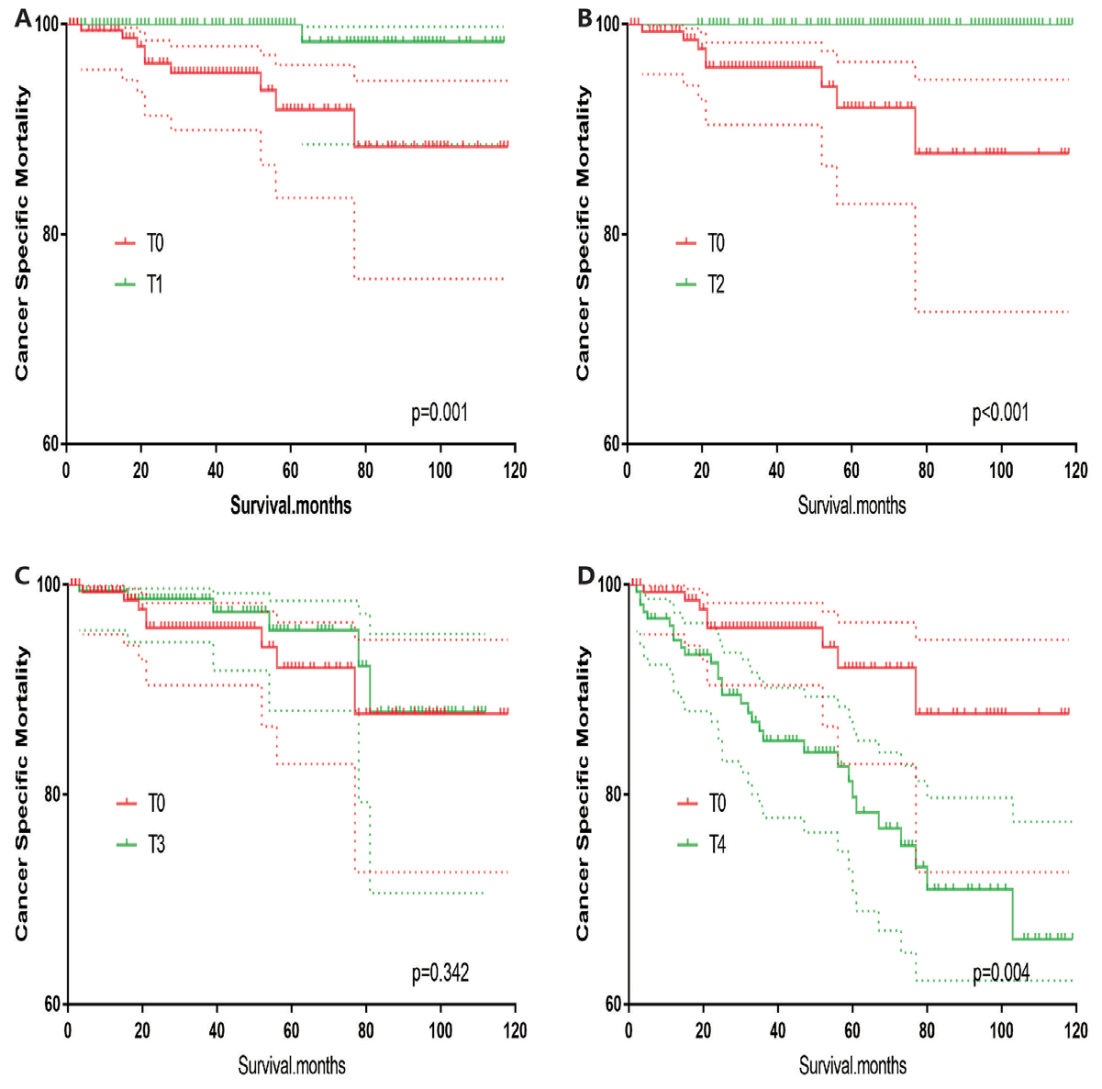
Kaplan Meier curves of cancer-specific mortality for matched T-stage pairs Age, sex and race matching between T0 and T1 **(A)**, T0 and T2 **(B)**, T0 and T3 **(C)**, T0 and T4 **(D)** patients.

**Figure 3:**
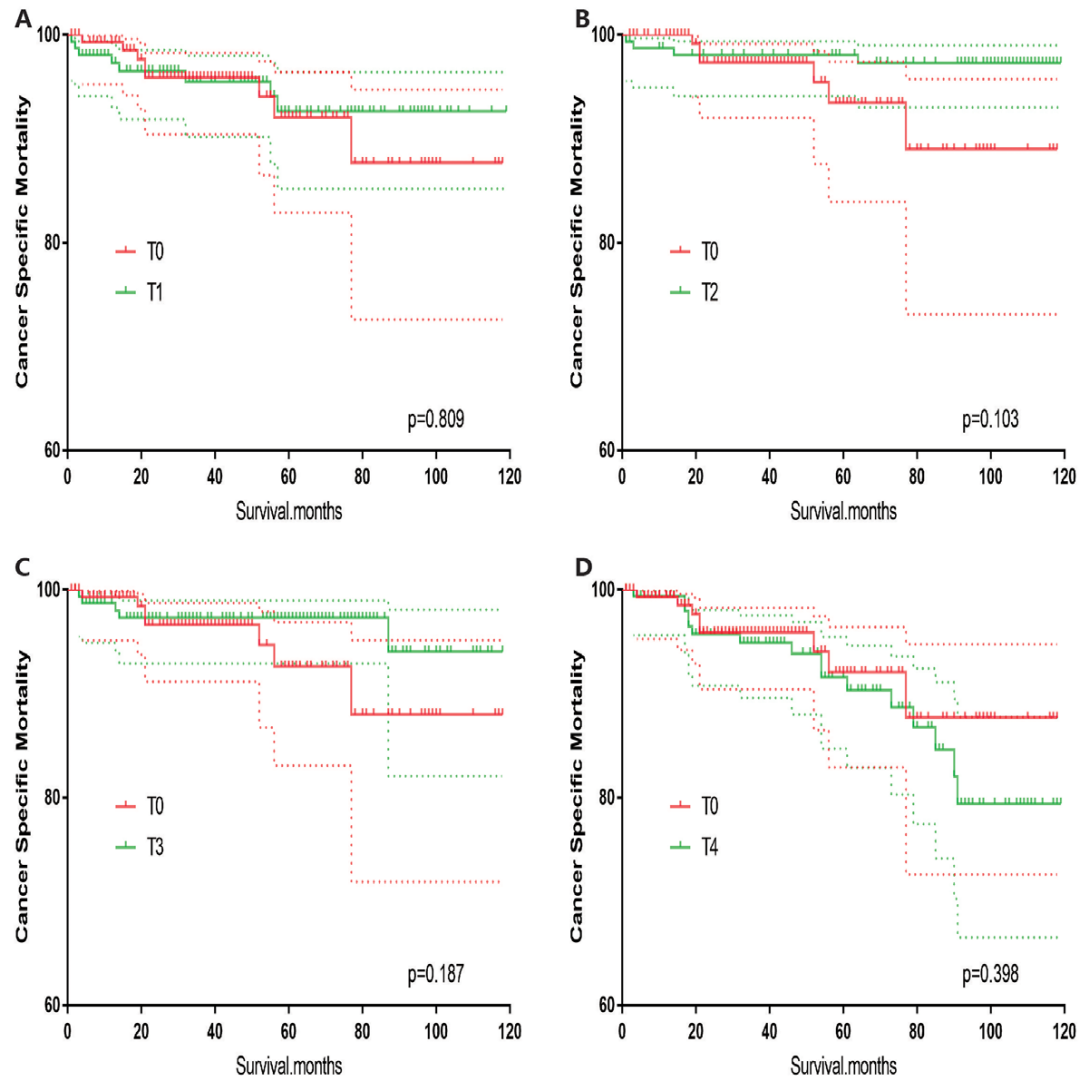
Kaplan Meier curves of cancer-specific mortality for matched T-stage pairs Age, sex, race, N/M stage, histologic subtype matched between T0 and T1 **(A)**, T0 and T2 **(B)**, T0 and T3 **(C)**, T0 and T4 **(D)** patients.

**Figure 4:**
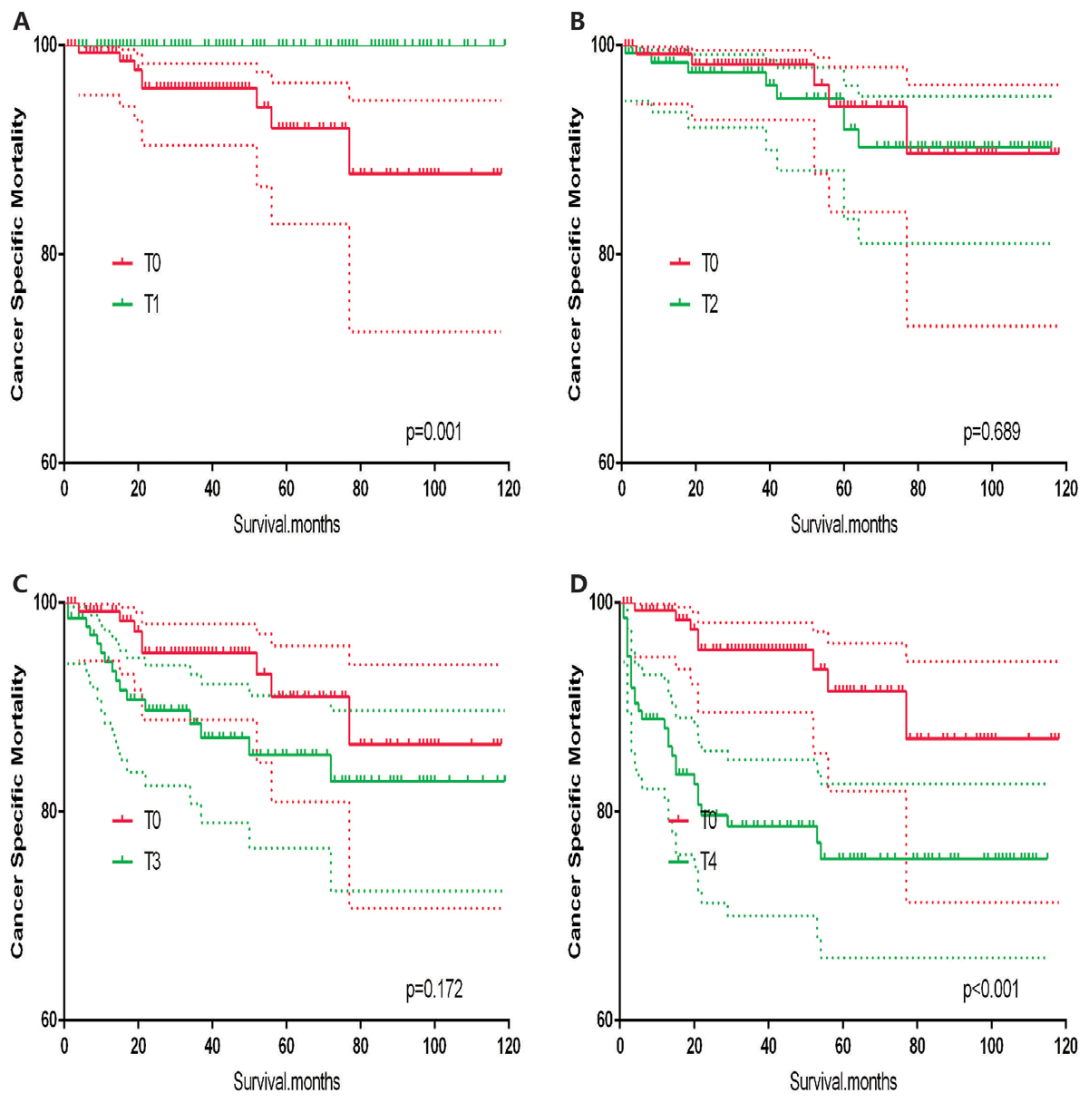
Kaplan Meier curves of cancer-specific mortality for matched T-stage pairs Age, sex, race, N/M stage, histologic subtype, surgery and radiation treatment matched between T0 and T1 **(A)**, T0 and T2 **(B)**, T0 and T3 **(C)**, T0 and T4 **(D)** patients.

T0 stage patients had a poorer prognosis (both cancer-specific mortality and all-cause mortality) compared to T1–T3 patients. However, they had a better prognosis than T4 stage patients for cancer-specific mortality, and similar with T4 stage patients for all-cause mortality (Figure 1A–1D). To minimize selection bias, propensity scored matching analysis was performed regarding the age, sex, race, N/M stage, histologic subtype, surgery and radiation treatment approaches. In survival analysis, T0 stage had a poorer prognosis for cancer-specific mortality compared to T1 and T2 stage (p=0.001,<0.001, respectively, Figure 2A, 2B), but T0 stage patients had a similar prognosis compared to T3 stage patients after propensity score matching for age, sex, and race. There was no significant difference between T0 stage and T1–T4 stages for cancer-specific mortality after propensity score matching for age, sex and race, N/M stage, and histologic subtype (Figure 3A–3D). After matching for all influential factors, including surgery and radiation treatment, T0 stage patients had a worse prognosis for cancer-specific mortality compared to T1 stage patients (p=0.001, Figure 4A) and better prognosis compared to T4 stage patients (p<0.001, Figure 4D) but not different with T2-T3 stage patients(p=0.689,0.172; respectively, Figure 4B, 4C).

**Figure 5:**
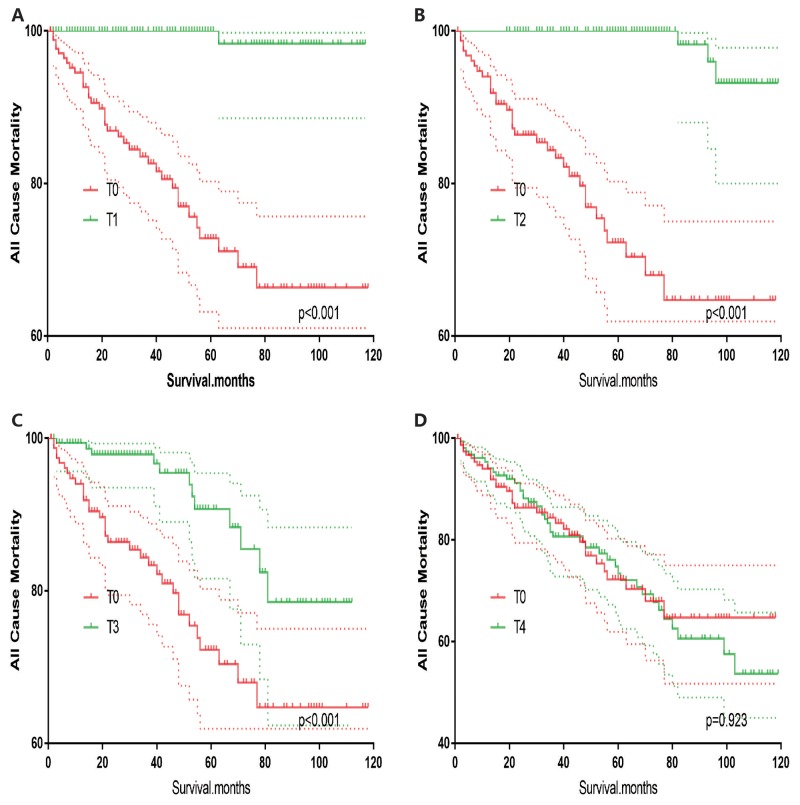
Kaplan Meier curves of all cause mortality for matched T-stage pairs Age, sex and race matching between T0 and T1 **(A)**, T0 and T2 **(B)**, T0 and T3 **(C)**, T0 and T4 **(D)** patients.

**Figure 6:**
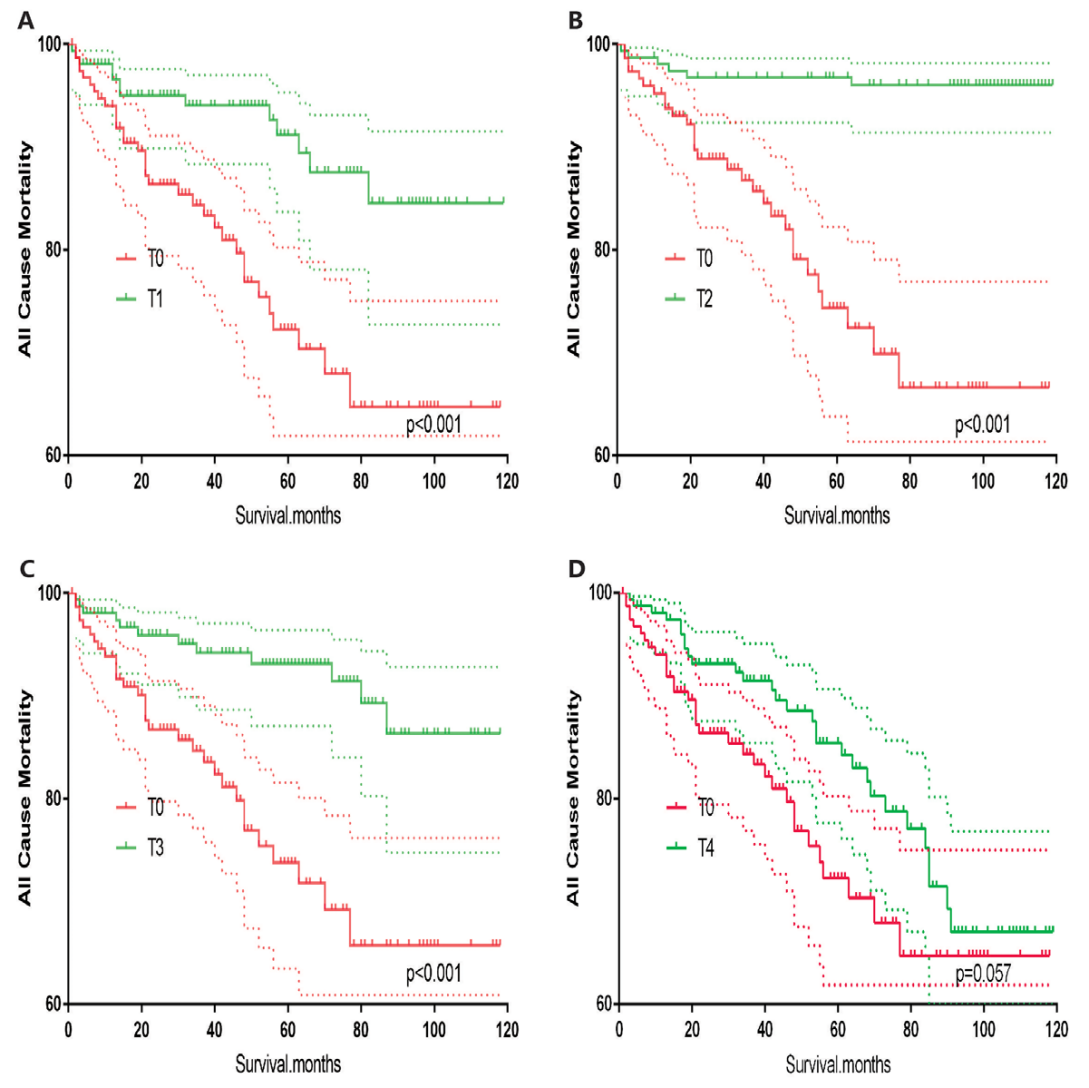
Kaplan Meier curves of all cause mortality for matched T-stage pairs Age, sex, race, N/M stage, histologic subtype matching between T0 and T1 **(A)**, T0 and T2 **(B)**, T0 and T3 **(C)**, T0 and T4 **(D)** patients.

**Figure 7:**
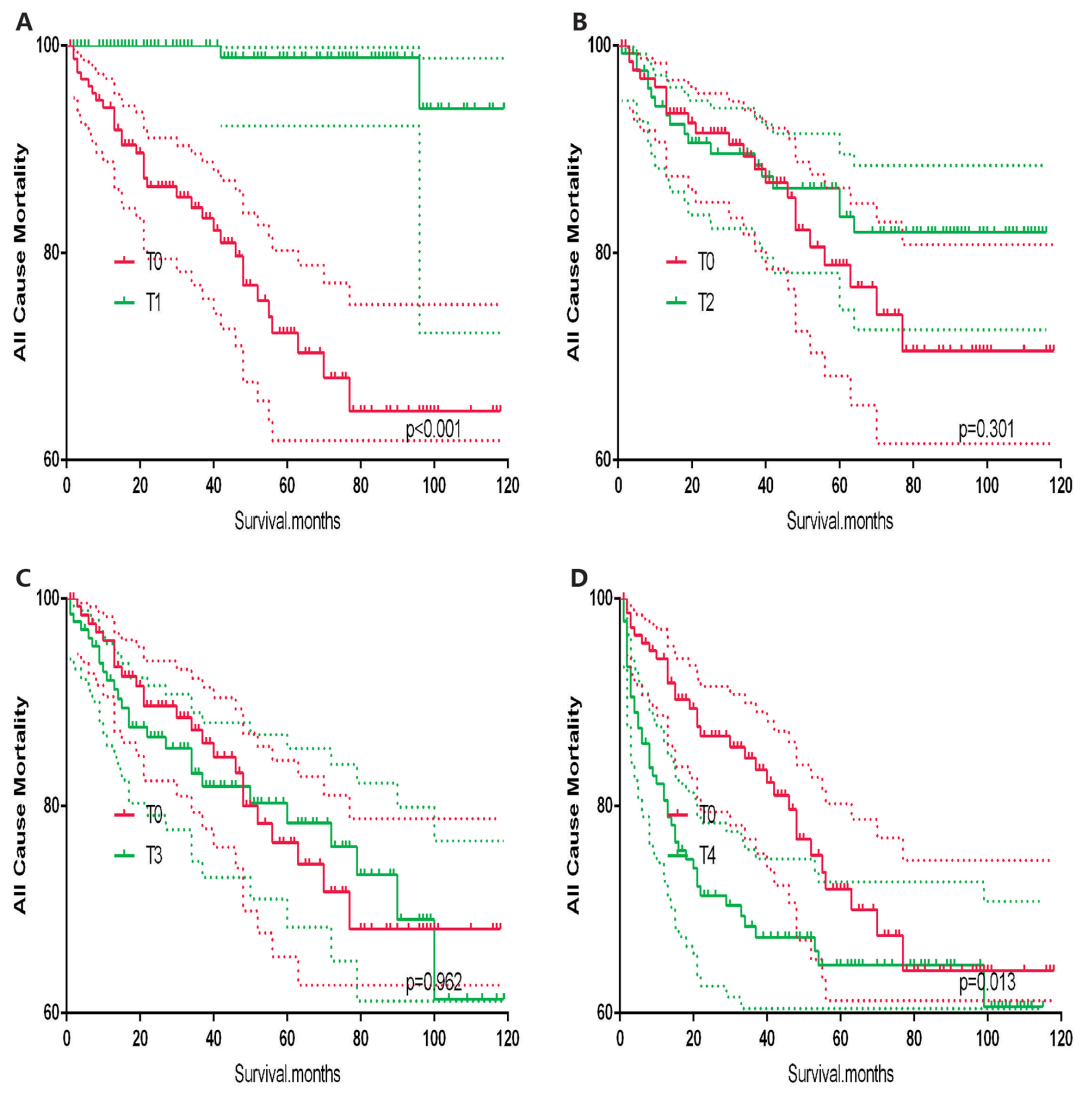
Kaplan Meier curves of all cause mortality for matched T-stage pairs Age, sex, race, N/M stage, histologic subtype, surgery and radiation treatment matching between T0 and T1 **(A)**, T0 and T2 **(B)**, T0 and T3 **(C)**, T0 and T4 **(D)** patients.

In survival analysis for all-cause mortality, T0 stage patients had a poorer prognosis compared to T1–T3 stage patients (all p<0.001, Figure 5A–5C), but similar prognosis as T4 stage patients after matching for age, sex and race (Figure 5D). Similar results were obtained after matching for age, sex and race, N/M stage, histologic subtype (Figure 6A–6D). After matching for all influential factors including surgery and radiation treatment, T0 stage patients showed a better prognosis for all-cause mortality compared to T1 stage patients (p<0.001, Figure 7A), but no difference when compared to T2–T4 patients (Figure 7B–7D).

## DISCUSSION

The previous and current edition (6th and 7th) of the AJCC guidelines defines T0 stage as there being no evidence of a primary tumor. However, the prognosis of T0 stage DTC patients was not thoroughly investigated in the literature. In this study, we evaluated the prognosis of T0 stage DTCs from the SEER database based on diagnoses from 2004–2013. We found that T0 stage patients had a poorer prognosis than expected after adjustment for influential risk factors.

One of the possible reasons for not finding evidence of a primary tumor, and hence a T0 stage diagnosis, is the tumor is too small for detection [11, 12]. The detection rates of papillary thyroid microcarcinoma (PTMC) and occult thyroid carcinoma have increased due to the worldwide use of high-resolution sonography and ultrasound-guided fine-needle aspiration biopsy (US-FNAB) [12, 13]. In particular, US-FNAB has been performed for small thyroid nodules regardless of the nodule size. Kim et al. extracted a PTMC sample with a 1 mm tumor size by US-FNAB [12]. Therefore, the primary tumor may not be detected when undergoing a thyroidectomy.

The balance between inadequate and excessive treatment is a pivotal concern in the management of thyroid carcinoma. Currently, no local surgery or radiation treatment being recommended for T0 stage patients may a consequence of the 2015 ATA guidelines for the management of thyroid nodules and DTC [6]. In addition, surgeons and oncologists choosing to the current management course for T0 patients may be due to the observed low-mortality rate [7, 10].

Postoperative radioiodine ablation treatment can eradicate normal-thyroid remnants to reduce or eliminate serum thyroglobulin levels, as well as irradiate neoplastic foci, thereby decreasing the risk of mortality and recurrence [14]. However, radioiodine may also induce lacrimal and salivary gland toxicities [15, 16]. At present, few studies have examined the administration of radioiodine to provide therapeutic benefits after a complete thyroidectomy. In our current study, only 41.1% of patients with T0 stage underwent radioactive I-131 ablation as compared with 60.5% with T2 stage, 66.1% with T3 stage, and 59.1% with T4 stage, according to the SEER database.

Nevertheless, our study demonstrated that 58.3% of patients underwent total thyroidectomy as compared to 76.3% with T1 stage, 82.4% with T2 stage, 88.4% with T3 stage, and 82.4% with T4 stage. Therefore, a conservative treatment approach may play an important role in DTCs’ mortality with T0 stage patients.

Other clinicopathological features, such as more aggressive tumor histologies, multifocality, lymph node metastasis, and extrathyroidal extension, may play a larger role than tumor size alone with regard to patient prognosis [17-20]. In this study, for example, lymph node metastasis accounts for 73.3% of T0 stage patients, but 11.9% of T1 stage patients had lymph node metastasis.

T0 patients had a higher incidence of distant metastasis (45/180, 25.0%) than any other T stages patients did in this study. According to previous studies, distant metastasis was a significant risk for thyroid cancer-specific mortality and all-cause mortality [17, 21]. Therefore, the high prevalence of distant metastasis in T0 stage may result in a higher mortality from DTC. Our hypothesis was also strengthened by the results from the propensity score matching analysis.

Our study had the following limitations. One limitation of this study is that the utilized dataset lacked information regarding recurrence, thereby introducing overestimation bias when designating cancer-specific death and all-cause death. Another limitation of this study is that family history, vascular invasion, and other histologic findings were not evaluated or included in our study. Furthermore, the molecular markers such as *BRAF* point mutation and *TERT* promoter point mutations were not observed in our study or adjusted for in our analyses.

In summary, T0 patients had a significantly poorer survival than T1–T3 patients. These results are not consistent with current expectations of DTC progression and raise new implications for the treatment of patients with T0 stage DTC.

## MATERIALS AND METHODS

### Study population

We investigated a large number of DTC patients from the SEER program. The SEER project is a United States population-based cancer registry that began in 1973, and is supported by both the National Cancer Institute and Centers for Disease Control and Prevention. It contains cancer data from across multiple geographic regions on the incidence, prevalence, mortality, population-based variables, primary tumor characteristics, and more.

### Data collection and analysis

We examined SEER data from 2004 to 2013 and selected patients with a diagnosis of DTC, as defined by a combination of ICD-O site code of C73.9 (i.e., thyroid, papillary, and/or follicular histology). The diagnosis codes were included in the study: “papillary carcinoma”, “papillary adenocarcinoma”, “oxyphilic adenocarcinoma”, “follicular adenocarcinoma”, “papillary & follicular adenocarcinoma”, and “papillary cyst-adenocarcinoma”. To compare the survival rate among different T stages, 94092 patients were categorized according to AJCC T staging (version 6 and 7, Supplementary Tables 1 and 2). Age, sex, race, N/M stage, histologic subtype, surgery (biopsy, lobectomy, subtotal or near-total thyroidectomy, and total thyroidectomy) and radiation (none or refused, external beam radiation therapy, and radioactive I-131 ablation) treatments were evaluated in patients with different T stages.

### Statistical analyses

Patients were followed-up until December 2013. Patient survival curves (thyroid cancer-specific mortality and all-cause mortality) were examined by Kaplan-Meier analyses with the log-rank test. To further adjust for potential baseline confounding factors, a propensity score matching analysis was conducted. Cox proportional hazards regression analyses were performed to estimate the hazard ratios with 95% Cis, to show the magnitude of the effect of stage on cancer-specific mortality and all-cause mortality [22]. All p-values were 2-sided, with p <.05 being considered significant. Analyses were performed using SPSS version 19.0, Stata/SE version 12 (Stata Corp.), and GraphPad Prism version 6 (GraphPad Software Inc.).

## SUPPLEMENTARY MATERIALS TABLES






