# Correction: Unanticipated prognosis of differential thyroid cancer patients with T0 stage: analysis of the SEER database 2004-2013

**DOI:** 10.18632/oncotarget.24999

**Published:** 2018-03-23

**Authors:** Chunping Liu, Jie Ming, Wen Zeng, Shuntao Wang, Yiquan Xiong, Qiuyang Zhao, Xingjie Yin, Zeming Liu, Tao Huang

**Affiliations:** ^1^ Department of Breast and Thyroid Surgery, Union Hospital, Tongji Medical College, Huazhong University of Science and Technology, Wuhan 430022, China; ^2^ Department of Ophthalmology, Zhongnan Hospital, Wuhan University, Wuhan, China

**This article has been corrected:** The proper name of the institution is as follows:

^1^ Department of Breast and Thyroid Surgery, Union Hospital, Tongji Medical College, Huazhong University of Science and Technology, Wuhan 430022, China

Original article: Oncotarget. 2017; 8:70777-70787. https://doi.org/10.18632/oncotarget.19988

