# Supplementary material for: Unanticipated prognosis of differential thyroid cancer patients with T0 stage: analysis of the SEER database 2004-2013
Source: Oncotarget. 2017 Aug 7;8(41):70777–87. doi: 10.18632/oncotarget.19988 (PMC5642593; doi:10.18632/oncotarget.19988)
Supplement: Supplementary file 1 [file oncotarget-08-70777-s001.pdf]

Unanticipated prognosis of differential thyroid cancer patients with T0 stage: analysis of the SEER database 2004-2013

SUPPLEMENTARY MATERIALS

Supplementary Table 1: AJCC Cancer Staging Manual, 6th Edition: protocol for differentiated thyroid carcinoma.

See Supplementary File 1

Supplementary Table 2: AJCC Cancer Staging Manual, 7th Edition: protocol for differentiated thyroid carcinoma.

See Supplementary File 2
