# Supplementary material for: Unanticipated prognosis of differential thyroid cancer patients with T0 stage: analysis of the SEER database 2004-2013
Source: Oncotarget. 2017 Aug 7;8(41):70777–87. doi: 10.18632/oncotarget.19988 (PMC5642593; doi:10.18632/oncotarget.19988)
Supplement: Supplementary file 2 [file oncotarget-08-70777-s002.docx]

**Supplementary Table 2.** **AJCC Cancer Staging Manual, 7th Edition: protocol for differentiated thyroid carcinoma**

| AJCC Staging Protocol for DTC, 7th Edition | | | |
| --- | --- | --- | --- |
| Primary tumor (T) | | | |
| TX Primary tumor cannot be assessed | | | |
| T0 No evidence of primary tumor | | | |
| T1 ≤ 2cm, limited to the thyroid | | | |
| T1a ≤ 1cm | | | |
| T1b > 1 cm and ≤ 2 cm | | | |
| T2 > 2 cm and ≤ 4 cm, limited to the thyroid | | | |
| T3 > 4 cm, limited to the thyroid or any tumor with  minimal extra-thyroid extension (e.g., to the  sternothyriod muscle or perithyroid soft tissues) | | | |
| T4a Tumor of any size extending beyond the thyroid  capsule to invade subcutaneous soft tissues, larynx,  trachea, esophagus, or recurrent laryngeal nerve | | | |
| T4b Tumor invades prevertebral fascia or encases  carotid artery or mediastinal vessels | | | |
| Regional nodes (N) | | | |
| NX Regional lymph nodes cannot be assessed | | | |
| N0 No regional node metastasis | | | |
| N1 Regional node involvement | | | |
| N1a Nodal metastasis to level VI (pretracheal, para-  tracheal, and prelaryngeal/Delphian lymph nodes) | | | |
| N1b Nodal metastasis to unilateral, bilateral, or contralateral cervical (Levels I, II, III, IV, or V) or  retropharyngeal or cervical or superior mediastinal  lymph nodes (Level VII) | | | |
| Distant metastasis (M) | | | |
| M0 No distant metastasis | | | |
| M1 Distant metastasis | | | |
| AJCC Staging grouping | | | |
| For patients < 45 years | | | |
| Stage I | Any T | Any N | M0 |
| Stage II | Any T | Any N | M1 |
| For patients≥ 45 years | | | |
| Stage I | T1 | N0 | M0 |
| Stage II | T2 | N0 | M0 |
| Stage III | T3 | N0 | M0 |
|  | T1–3 | N1a | M0 |
| Stage IVa | T4a | N0–1a | M0 |
|  | T1–4a | N1b | M0 |
| Stage IVb | T4b | Any N | M0 |
| Stage IVc | Any T | Any N | M1 |
