# Supplementary material for: Unanticipated prognosis of differential thyroid cancer patients with T0 stage: analysis of the SEER database 2004-2013
Source: Oncotarget. 2017 Aug 7;8(41):70777–87. doi: 10.18632/oncotarget.19988 (PMC5642593; doi:10.18632/oncotarget.19988)
Supplement: Supplementary file 3 [file oncotarget-08-70777-s003.docx]

**Supplementary Table 1: AJCC Cancer Staging Manual, 6th Edition: protocol for differentiated thyroid carcinoma**

| AJCC Staging Protocol for DTC, 6th Edition | | | |
| --- | --- | --- | --- |
| Primary tumor (T)  Note: All categories may be subdivided: (a) solitary tumor, (b) multifocal (the largest determines the classification). | | | |
| TX Primary tumor cannot be assessed | | | |
| T0 No evidence of primary tumor | | | |
| T1 Tumor 2 cm or less in greatest dimension limited to the thyroid | | | |
| T2 Tumor more than 2 cm but not more than 4 cm in greatest dimension limited to the thyroid | | | |
| T3 Tumor more than 4 cm in greatest dimension limited to the thyroid or any tumor with  minimal extrathyroid extension (e.g., extension to the sternothyriod muscle or perithyroid soft tissues) | | | |
| T4a Tumor of any size extending beyond the thyroid capsule to invade subcutaneous soft tissues, larynx, trachea, esophagus, or recurrent laryngeal nerve | | | |
| T4b Tumor invades prevertebral fascia or encases carotid artery or mediastinal vessels | | | |
| Regional nodes (N)  Regional lymph nodes are the central compartment, lateral cervical, and upper mediastinal lymph nodes. | | | |
| NX Regional lymph nodes cannot be assessed | | | |
| N0 No regional node metastasis | | | |
| N1 Regional node involvement | | | |
| N1a Nodal metastasis to level VI (pretracheal, paratracheal, and prelaryngeal/Delphian lymph nodes) | | | |
| N1b Metastasis to unilateral, bilateral, or contralateral cervical or cervical or superior mediastinal lymph nodes | | | |
| Distant metastasis (M)  MX Distant metastasis cannot be assessed | | | |
| M0 No distant metastasis | | | |
| M1 Distant metastasis | | | |
| AJCC Staging grouping | | | |
| For patients < 45 years | | | |
| Stage I | Any T | Any N | M0 |
| Stage II | Any T | Any N | M1 |
| For patients≥ 45 years | | | |
| Stage I | T1 | N0 | M0 |
| Stage II | T2 | N0 | M0 |
| Stage III | T3 | N0 | M0 |
|  | T1 | N1a | M0 |
|  | T2 | N1a | M0 |
|  | T3 | N1a | M0 |
| Stage IVa | T4a | N0 | M0 |
|  | T4a | N1a | M0 |
|  | T1 | N1b | M0 |
|  | T2 | N1b | M0 |
|  | T3 | N1b | M0 |
|  | T4a | N1b | M0 |
| Stage IVb | T4b | Any N | M0 |
| Stage IVc | Any T | Any N | M1 |
